# Truancy and teenage pregnancy in English adolescent girls: can we identify those at risk?

**DOI:** 10.1093/pubmed/fdv029

**Published:** 2015-03-16

**Authors:** Yin Zhou, Dewi Ismajani Puradiredja, Gary Abel

**Affiliations:** 1 The Primary Care Unit, Department of Public Health and Primary Care, University of Cambridge, Cambridge CB2 0SR, UK; 2 Department of Population Health, London School of Hygiene and Tropical Medicine, London, UK

**Keywords:** adolescents, sexual behaviour, social determinants, teenage pregnancy, truancy, young people

## Abstract

**Background:**

Truancy has been linked to risky sexual behaviours in teenagers. However, no studies in England have examined the association between truancy and teenage pregnancy, and the use of truancy as a marker of teenagers at risk of pregnancy.

**Methods:**

Using logistic regression, we investigated the association between truancy at age 15 and the likelihood of teenage pregnancy by age 19 among 3837 female teenagers who participated in the Longitudinal Study of Young People of England. We calculated the areas under the ROC curves of four models to determine how useful truancy would be as a marker of future teenage pregnancy.

**Results:**

Truancy showed a dose–response association with teenage pregnancy after adjusting for ethnicity, educational intentions at age 16, parental socioeconomic status and family composition (‘several days at a time’ versus ‘none’, odds ratio 3.48 95% confidence interval 1.90–6.36, *P* < 0.001). Inclusion of risk behaviours improved the accuracy of predictive models only marginally (area under the ROC curve 0.76 full model versus 0.71 sociodemographic characteristics only).

**Conclusions:**

Truancy is independently associated with teenage pregnancy among English adolescent girls. However, the discriminatory powers of models were low, suggesting that interventions addressing the whole population, rather than targeting high-risk individuals, might be more effective in reducing teenage pregnancy rates.

## Introduction

Despite significant declines in under 18 birth rates in the last decade,^1^ the UK remains the country with the highest teenage pregnancy rate in Western Europe.^2^ In 2012, the under-18 conception rate for England was 27.9 conceptions per 1000 girls aged 15–17.^3^ Teenage pregnancy not only puts the young mother at risk of adverse health effects, but also has socioeconomic implications for the teenage parents, their children and society at large.^4^ For instance, teenage conception is linked with spontaneous and induced abortions, sexually transmitted infections, as well as neonatal and maternal mortality and morbidity.^4,5^ In the UK, teenage mothers are six times more likely to live in social housing, four times more likely to be in a family where neither of the couple is employed, and three times as likely to be on government welfare support as older mothers by the age of 30.^6^

Teenage pregnancy has largely been viewed as a negative phenomenon in developed countries. The British government has made reducing the under 18 conception rate one of the top priorities in promoting adolescent health.^7,8^ Teenage pregnancy may be a result of risky sexual behaviour, which tends to co-occur with other risk behaviours^9^ such as truancy.^10–12^ While levels of overall absence across all maintained schools have dropped from 6.3 to 5.2% between 2008/09 and 2012/13 in the UK, unauthorized absences (or truancy) have maintained around 1.0%.^13^ To our knowledge, there are no studies thus far examining the association between truancy and teenage pregnancy using cohort survey designs. Using data from different survey waves of the Longitudinal Study of Young People in England (LSYPE), we aimed to determine whether truancy at age 15 is associated with teenage pregnancy before age 19, and whether truancy, an observable risk behaviour, can be a useful marker of teenage pregnancy.

## Methods

### Sample

The analyses of this paper draw on data collected as part of the LSYPE—a prospective cohort study that followed a nationally representative sample of around 15 500 English adolescents (born between 1 September 1989 and 31 August 1990) throughout their teenage years. The study was commissioned by the former Department for Education and Skills (DfES) and now managed by the Department for Education (DfE). The anonymized records and LSYPE data sets exist in the public domain (via the UK Data Service), and ethical clearance has been obtained by the DfE from an in-country panel in accordance with the ESRC Research Ethics Framework.^14^ It is not possible to identify individuals from the information provided.

The complete description of the design of the LSYPE has been published elsewhere.^15^ The baseline survey (Wave 1) was carried out in 2004, participants were aged between 13 and 14, and was repeated annually until 2010. For convenience, age references will be made with respect to the upper end of the range for each wave in subsequent text (Table 1). Response rates ranged from 74 to 92% across all the waves. Our analysis sample was restricted to female respondents who had complete data on teenage pregnancy in Wave 6, all risk behaviours and other sociodemographic variables (*n* = 3837). Risk behaviour data including frequency of truancy, alcohol consumption and cannabis use were drawn from Wave 3, when the adolescents were in their last year of compulsory education in the British education system (age 15–16) to reflect risk behaviours during school years. Pregnancy data were obtained from Wave 6, corresponding to age 18–19 in this cohort of adolescents.

**Table 1:** LSYPE study waves and corresponding ages of adolescents

*Survey wave (year of LSYPE)*	*Age range of adolescents*	*Age referred to in text*
1 (2004)	13–14	14
2 (2005)	14–15	15
3 (2006)	15–16	16
4 (2007)	16–17	17
5 (2008)	17–18	18
6 (2009)	18–19	19

### Measures

We distinguished girls who have ever been pregnant from those who have not by dichotomizing the outcome variable into two groups. Because pregnancy status was only asked of those who reported having had sex, teenage girls who reported to have never had sex or who reported to have never been pregnant at age 19 were grouped into one, and those who had reported to have ever been pregnant the other.

Truancy data were obtained from age 16, when participants were asked, ‘Since the last time we spoke to you in (text fill: Wave 2 interview month) last year, (have/did) you (played/play) truant, that is missed school without permission, even if it was only for a half day or a single lesson?’. The truancy data therefore represented this risk behaviour in the year prior to being interviewed at age 16 (i.e., at age 14–15). Respondents were subsequently asked the frequency of playing truant if they replied ‘yes’ to the screening question (Supplementary data, Appendix 1).

Other variables adjusted for in our analyses include ethnicity, future educational intentions, parental socioeconomic status and family composition at age 16, as previously described predictors of teenage pregnancy.^16–24^ Further, because risk behaviours tend to coexist, we also explored whether the effect of truancy on teenage pregnancy was affected by other common risk behaviours undertaken by teenage girls. We therefore included data on frequency of alcohol consumption and ever use of cannabis, the commonest drug used by adolescents in the UK,^25^ in our all adjusted model.

### Statistical analyses

Descriptive statistics were produced to compare frequency distributions of analysis across all variables, stratified by teenage pregnancy status. Subsequently, associations of teenage pregnancy status with other variables were estimated using logistic regression. Firstly, we estimated the crude associations between teenage pregnancy and all other variables. We then constructed an adjusted model including truancy at age 15, sociodemographic and educational variables, to see whether truancy is associated with teenage pregnancy after accounting for sociodemographic and educational factors. A fully adjusted model then augmented the adjusted model with two further risk behaviours (alcohol consumption and cannabis use) to see whether any association between teenage pregnancy and truancy persisted after adjustment for these variables. Finally, to assess the predictive usefulness of truancy as a marker of future teenage pregnancy, we produced receiver operator curves (ROC). Here we treat a prediction based on the models described above as a diagnostic test of future teenage pregnancy. This was done for the two adjusted models described above as well as a model with only sociodemographic and educational variables, and a model that contained sociodemographic and educational variables plus alcohol consumption and cannabis use. Comparison of these ROC curves enabled additional assessment of the discriminatory power of truancy at age 15.

All statistical analyses were conducted using STATA v12.

## Results

Of the 3837 girls in the sample, 433 (11.3%) reported having ever been pregnant before or at 19 years old. Eight hundred and fifty-one (22.2%) reported having ever played truant at age 15.

In the crude model, we found strong evidence (*P* < 0.001) of a dose–response relationship between frequency of truancy and likelihood of teenage pregnancy (Table 2). For example, teenage girls who played truant ‘on the ‘odd day or lesson’ were more likely to have been pregnant than girls who did not play truant (odds ratio (OR) 1.98, 95% confidence interval (CI) 1.54–2.55), but less likely than those who played truant ‘several days at a time’ (OR compared with ‘Never’ 5.42, 95% CI 3.06–9.58). Teenage girls who had tried cannabis and those who drank alcohol were also more likely to report having been pregnant than their cannabis- and alcohol-free counterparts. Those girls who were intending to leave full-time education after 16 had lower parental socioeconomic status and with no parents in the family were also more likely to report teenage pregnancy at age 19.

**Table 2:** Frequency distribution and odds ratios of teenage pregnancy across all variables

*Variable*	*Response category*	*Number of teenagers*	*Number reporting pregnancy (%)*	Unadjusted model*		Adjusted model**		All adjusted model***	
				*OR (95% CI)*	**P*-value*	*OR (95% CI)*	**P*-value*	*OR (95% CI)*	**P*-value*
Frequency of truancy (*n* = 3837)	None	2986	265 (8.87)	Ref	<0.001	Ref	<0.001	Ref	0.0331
	Odd day or lesson	588	95 (16.16)	1.98 (1.54–2.55)		1.69 (1.30–2.21)		1.16 (0.87–1.55)	
	Particular lessons	175	42 (24)	3.24 (2.24–4.69)		2.64 (1.79–3.91)		1.72 (1.14–2.61)	
	Several days at a time	55	19 (34.55)	5.42 (3.06–9.58)		3.48 (1.90–6.36)		2.05 (1.08–3.92)	
	Weeks at a time	33	12 (36.36)	5.87 (2.85–12.06)		3.12 (1.40–6.97)		1.67 (0.71–3.90)	
Ethnicity (*n* = 3837)	White	2743	340 (12.40)	Ref	<0.001	Ref	<0.001	Ref	0.0061
	Mixed	181	29 (16.02)	1.35 (0.89–2.04)		1.04 (0.67–1.62)		0.99 (0.63–1.56)	
	Asian: Indian/Pakistani/Bangladeshi	601	20 (3.33)	0.24 (0.15–0.39)		0.24 (0.15–0.40)		0.40 (0.23–0.68)	
	Black: Black Carribean, Black African	209	33 (15.79)	1.33 (0.90–1.95)		0.99 (0.64–1.51)		1.24 (0.79–1.93)	
	Other	103	11 (10.68)	0.85 (0.45–1.60)		0.72 (0.37–1.39)		0.89 (0.45–1.78)	
Educational intentions at 16 (*n* = 3837)	Stay on in FTE	3585	352 (9.82)	Ref	<0.001	Ref	<0.001	Ref	<0.001
	Leaving FTE but returning later	185	63 (34.05)	4.74 (3.43–6.55)		3.40 (2.39–4.83)		3.19 (2.22–4.59)	
	Leaving FTE	13	5 (38.46)	5.74 (1.87–17.64)		4.46 (1.34–14.77)		4.41 (1.28–15.21)	
	Don't know	54	13 (24.07)	2.91 (1.55–5.49)		2.26 (1.16–4.40)		2.51 (1.27–4.95)	
SES (*n* = 3837)	1st quintile (of cohort)	945	79 (8.36)	Ref	<0.001	Ref	<0.001	Ref	<0.001
	2nd quintile	756	68 (8.99)	1.08 (0.77–1.52)		1.03 (0.73–1.47)		1.02 (0.71–1.44)	
	3rd quintile	718	78 (10.86)	1.34 (0.96–1.86)		1.13 (0.80–1.59)		1.19 (0.83–1.68)	
	4th quintile	731	107 (14.64)	1.88 (1.38–2.56)		1.74 (1.25–2.42)		1.88 (1.34–2.64)	
	5th quintile	687	101 (14.70)	1.89 (1.38–2.58)		1.93 (1.37–2.73)		2.15 (1.51–3.05)	
Family composition (*n* = 3837)	Married couple	2715	214 (7.88)	Ref	<0.001	Ref	Ref <0.001	Ref	<0.001
	Cohabiting couple	247	51 (20.65)	3.04 (2.17–4.26)		2.11 (1.47–3.01)		2.06 (1.43–2.95)	
	Lone father	54	16 (29.63)	4.92 (2.70–8.97)		3.41 (1.81–6.42)		3.29 (1.71–6.34)	
	Lone mother	797	143 (17.94)	2.56 (2.03–3.21)		1.84 (1.44–2.36)		1.77 (1.37–2.28)	
	No parent	24	9 (37.50)	7.01 (3.03–16.21)		7.42 (3.08–17.90)		7.83 (3.17–19.29)	
Alcohol frequency (*n* = 3837)	Never	1161	67 (5.77)	Ref	<0.001	–	–	Ref	0.0064
	Less often than once every couple of months	539	56 (10.39)	1.89 (1.31–2.74)				1.30 (0.86–1.96)	
	Once every couple of months	567	67 (11.82)	2.19 (1.53–3.12)				1.72 (1.15–2.59)	
	Once a month	359	51 (14.21)	2.70 (1.84–3.98)				1.98 (1.27–3.09)	
	Two or three times a month	672	87 (12.95)	2.43 (1.74–3.39)				1.56 (1.03–2.35)	
	Once or twice a week	480	79 (16.46)	3.22 (2.28–4.54)				1.61 (1.03–2.51)	
	Most days	59	26 (44.07)	12.86 (7.27–22.75)				3.46 (1.74–6.87)	
Ever had cannabis (*n* = 3837)	No	2988	243 (8.13)	Ref		–	–	Ref	<0.001
	Yes	849	190 (22.38)	3.26 (2.65–4.01)	<0.001			2.00 (1.54–2.61)	

OR, odds ratio.

*Estimated from unadjusted analysis between each individual exposure or sociodemographic variable and teenage pregnancy; *P* < 0.001 for all association (joint tests for categorical variables).

**Estimated from one multivariate model adjusted for truancy, ethnicity, educational intentions at 15, socioeconomic status and family composition; *P* < 0.001 for all associations (joint Wald tests for categorical variables).

***Estimated from one multivariate model adjusted for all risk behaviours (truancy, alcohol and cannabis use), ethnicity, educational intentions at 16, socioeconomic status and family composition; *P* < 0.05 for all associations (joint Wald tests for categorical variables).

In the adjusted model, there remained strong evidence (*P* < 0.001) that having ever played truant at 15 was still associated with increased odds of ever being pregnant before 19 years of age after adjusting for ethnicity, future educational intentions at age 16, parental socioeconomic status and family composition. This association was weaker than the crude association (e.g., ‘several days at a time’ OR 3.48 95% CI 1.90–6.36), but the dose–response relationship remained.

In the fully adjusted model, we further accounted for alcohol consumption and cannabis use. While the strength of the association between truancy at 15 and an increased likelihood of self-reported pregnancy at 19 became weaker after adjustment (*P* = 0.033), this relationship was attenuated compared with that in the previous two models (e.g., ‘several days at a time’ OR 2.05 95% CI 1.08–3.92). Further the dose–response relationship between truancy and pregnancy was no longer clear. These findings imply that truant teenagers who became pregnant were also more likely to consume alcohol and have tried cannabis.

## Is truancy a useful marker of teenage pregnancy?

**Fig. 1:**
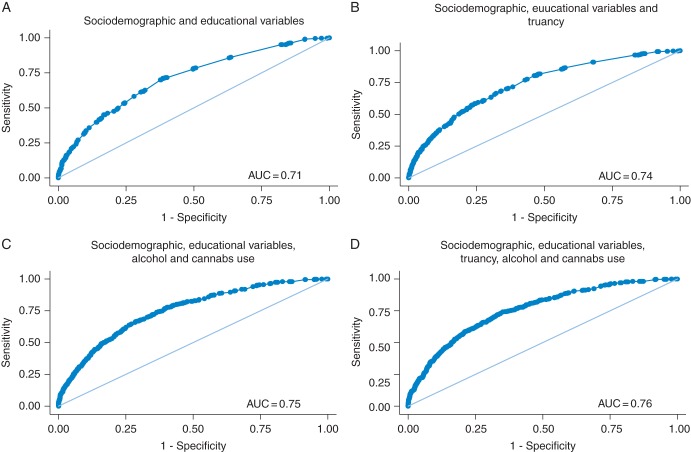
ROC curves for four models.

Figure 1 shows the ROC curves for each of the four models (with its included predictors) considered (see Section Methods). The area under the ROC curve (AUC) gives an indication of how well each model would perform when used to identify girls who will and those who will not report pregnancy at 19. A value tending towards 1 shows higher discriminatory power of the model, i.e. correctly distinguishing girls who will report having been pregnant from those who will not.

The areas in each of the ROC curves increased with each model. However, all four curves had areas <0.8, a cut-off value that is generally used for models with good discriminatory power.^26^ While the frequency of truancy increased the discriminatory power of the model with sociodemographic variables (Fig. 1B AUC 0.74 versus Fig. 1A AUC 0.71), alcohol and cannabis use were stronger predictors of teenage pregnancy than truancy (Fig. 1C AUC 0.75 versus Fig. 1B 0.74). As expected, the model with all predictor variables demonstrated the strongest predictive power (Fig. 1D AUC 0.76). However, it should be noted that the addition of any markers of risk behaviour (truancy, alcohol and cannabis use) provided only modest increases in AUC values.

Finally, it is useful to consider what sensitivity and specificity these models can achieve using different cut points. For example, to achieve a sensitivity of 0.5, a model using sociodemographic and educational variables and truancy (Fig. 1B) would achieve a specificity of ∼0.81. In other words to identify half of all 15-year-old girls who will have had a teenage pregnancy by age 19, over 19% of girls who do not get pregnant will also be flagged as having a higher risk of teenage pregnancy. To achieve a higher sensitivity of 0.75 (i.e. identifying three-quarters of teenage pregnancies), a specificity of only 0.57 is achieved. When also including alcohol and cannabis use in the model, these numbers only improve marginally to 0.84 and 0.62 (for sensitivities of 0.5 and 0.75, respectively—Fig. 1D).

## Discussion

### Main finding of this study

Our findings indicate that English girls who played truant at age 15 were more likely to have been pregnant before age 19, after accounting for ethnicity, educational intention at age 16, parental socioeconomic status and family composition. This association is attenuated when adjustments are made for other risk behaviours such as frequency of alcohol consumption and cannabis use. We also analysed the usefulness of truancy as a marker of teenage pregnancy and found that this is a weaker predictor than other risk behaviours such as alcohol and cannabis use.

### What is already known on this topic

This study builds on previous evidence that education-related ‘attitudes’ such as poor school ethos and school disaffection are linked to teenage pregnancy.^27–31^ However, this is the first to use a representative sample to explore the association between truancy, an observable risk ‘behaviour’, and teenage pregnancy in England. Our findings may inform the development of teenage pregnancy interventions targeting at-risk individuals. The attenuation of the association between truancy and teenage pregnancy in the all-adjusted model suggests that truancy, alcohol and cannabis use may coexist in those individuals who are more likely to be pregnant. This corroborates previous evidence that risk behaviours tend to co-occur^9^ and may represent an underlying trait that these individuals have that may increase their risk of teenage pregnancy. For example, truant adolescents have been found to be at increased risk of early sexual debut and can have concurrent sexual partners and sexual intercourse under the influence of drugs.^32–34^ At a programmatic level, targeting one risk behaviour (such as truancy) is unlikely to be effective in reducing teenage pregnancy.

### What this study adds

Strengths of our study include the use of a representative sample across England, high response rates during each wave, the ability to analyse individual-level data and the adjustment of known risk factors for pregnancy in the current literature. This is also the first English study to look at the direct link between truancy and teenage pregnancy in a cohort design setting, and the use of modelling to determine whether truancy can be used as a marker for the latter.

### Limitations of this study

The findings, however, should be considered with certain limitations in mind. Firstly, this study relies on retrospective self-reported behavioural data, and responses are therefore susceptible to recall bias. Secondly, sensitive behaviours, such as pregnancy, truancy, and drug and alcohol use, may be subject to under-reporting to produce answers judged to be socially desirable, although the ability to choose between face-to-face interviews and computerized self-administered questionnaires should have reduced the potential for biases to occur as a result of this. Further, due to data limitations, our measures of sexual and contraceptive behaviour do not permit differentiation between pregnancies that occurred before the age of 15 from those after. However, the majority of teenage pregnancy occurs above the age of 15 in the UK, with the age 13–15 conception rate being 5.6 per 1000 girls in 2012 (20% of that of the age group 15–17).^3^ Finally, the sample used in this study is solely female based, which prevented the opportunity to corroborate responses on sexual behaviour and to explore the effects of alcohol consumption from the perspective of male teenagers. We also note that our predictive models were not assessed against an independent validation data set and so should be considered over fitted and represent a best case scenario.

## Conclusions

This paper substantiates previous evidence on the association between persistent absenteeism from school and teenage pregnancy.^12^ Frequent truants who are at increased risk of teenage pregnancy may represent a group of vulnerable girls who may benefit from better psychological and social support to modify their overall risk behaviours. Our study found an independent association between truancy at 15 and teenage pregnancy before 19, with an attenuation of this relationship after adjusting for alcohol and cannabis use. While there is an association between risk behaviours (including truancy) and teenage pregnancy, these behaviours add little to the predictive power of models that could be used to identify individuals at risk of teenage pregnancy. Furthermore, such models, with or without risk behaviours, do not perform well at discriminating those at high and low risk. The implication of these findings is that interventions to reduce teenage pregnancy rates should be implemented at the population level rather than attempting to identify and target those at high risk.

## Supplementary data

Supplementary data are available at the *PUBMED* online.

## Funding

The work by Y.Z. is supported by an Academic Clinical Fellowship awarded by Health Education East of England (HEEoE). The work by D.I.P. is supported by the Economic and Social Science Research Council (ESRC) Grant ES.J004898.1.

## Authors' contributions

Y.Z. planned, analysed and drafted the article. D.I.P. and G.A. planned and commented on the article.

## Supplementary Material

Supplementary Data
