# Supplementary material for: Truancy and teenage pregnancy in English adolescent girls: can we identify those at risk?
Source: J Public Health (Oxf). 2015 Mar 16;38(2):323–9. doi: 10.1093/pubmed/fdv029 (PMC4894480; doi:10.1093/pubmed/fdv029)
Supplement: Supplementary Data [file supp_38_2_323__index.html]

Truancy and teenage pregnancy in English adolescent girls: can we identify those at risk? — Supplementary Data 

# Truancy and teenage pregnancy in English adolescent girls: can we identify those at risk?

## Supplementary Data

Supplementary Data

**Files in this Data Supplement:**

- Supplementary Data - Docx file
