# Supplementary material for: Truancy and teenage pregnancy in English adolescent girls: can we identify those at risk?
Source: J Public Health (Oxf). 2015 Mar 16;38(2):323–9. doi: 10.1093/pubmed/fdv029 (PMC4894480; doi:10.1093/pubmed/fdv029)
Supplement: Supplementary Data [file supp_fdv029_fdv029supp.docx]

**Appendix 1**

**Questions as they appear in LSYPE**

| Variable | Wave of LSYPE | Question |
| --- | --- | --- |
| Sexually active | 6 | Have you ever had sexual intercourse with someone?  1. Yes  2. No  Don’t know  Don’t want to answer |
| Pregnancy | 6 | (If yes to above question)  Have you ever been pregnant?   1. Yes 2. No 3. Don’t know 4. Don’t want to answer |
| Truancy | 3 | Since the last time we spoke to you in **(text fill: Wave 2 interview month) last year**, (have/did) you (played/play) truant, that is missed school without permission, even if it was only for a half day or a single lesson?  TYPE IN NUMBER AND THEN ENTER TO CONTINUE  1. Yes  2. No  3. Don't know  4. Don't want to answer |
| Frequency of truancy | 3 | What was the longest time (you've done/you did) this for since **(text fill: Wave 2 interview month)** last year? Was it...  TYPE IN NUMBER AND THEN ENTER TO CONTINUE  1. For weeks at a time  2. For several days at a time  3. For particular lessons  4. Only the odd day or lesson  8. Don't know  9. Don't want to answer |
| Ethnicity | 4 (derived from W1) | Description: This derivation takes the self-reported ethnicity of the Young Person from Wave 1 and updates it with any new  information given at wave 4 (this is also self-reported unless not given, when it is taken from the household grid). The new  information is primarily for the Wave 4 boost sample.  1 White  2 Mixed  3 Indian  4 Pakistani  5 Bangladeshi  6 Black Caribbean  7 Black African  8 Other |
| Future educational plans post Year 11 | 3 (Derived from 2 other questions in W3) | Description: This variable specifies the young person’s future educational plans. Depending on whether the interview took  place before or after the YP had finished year 11, respondents were either asked "When you have finished year 11 at  school what are you planning on doing?" (W3Plann16YP) or "Are you planning to go back into full time education in  September this year?" (W3RetEdYP). This variable combines the responses from these two questions.  1 “Staying on in /returning to FT education”  2 “Leaving /not returning to FT education”  3 “(SPONTANEOUS ONLY) Leaving/not returning to FT education now, but returning later”  -1 “Don’t know” |
| Family composition | 3 (Derived from other W3 variables) | This derivation uses the variables W3mother and W3father to identify the family composition of the (natural,  step, adoptive or foster) parents to the young person.  1 "Married couple"  2 "Cohabiting couple"  3 "Lone father"  4 "Lone mother"  5 "No parents in the household". |
| Alcohol consumption | 3 | Have you ever had a proper alcoholic drink? That is a whole drink, not just a sip. Please do not count drinks labelled low alcohol.  TYPE IN NUMBER AND THEN ENTER TO CONTINUE  1. Yes  2. No  8. Don't know  9. Don't want to answer  (If yes)  Thinking about the last 12 months, about how often did you usually have an alcoholic drink? Was it...  TYPE IN NUMBER AND THEN ENTER TO CONTINUE  1. Most days  2. Once or twice a week  3. 2 or 3 times a month  4. Once a month  5. Once every couple of months or  6. Less often?  8. Don't know  9. Don't want to answer |

| Cannabis use | 3 | The next question is about Cannabis, also called Marijuana, Dope, Pot, Blow, Hash, Skunk, Puff, Grass,  Draw, Ganja, Spliff, Joints, Smoke, Weed. Remember your name is not on this questionnaire so no-one who knows you will find out your answer.  1. Press <Enter> to continue.  Have you ever tried Cannabis even if only once?  TYPE IN NUMBER AND THEN ENTER TO CONTINUE  1. Yes  2. No  3. Don't know  4. Don't want to answer |
| --- | --- | --- |
